# Just Another Stroke? A Case of Infective Endocarditis Causing an Embolic Stroke and Splenic Aneurysms

**DOI:** 10.5334/jbr-btr.957

**Published:** 2016-01-28

**Authors:** Sinead Culleton

**Affiliations:** 1 Galway University Hospital, IE

A 35 year old male presented to the emergency department with a four day history of a right sided facial droop and expressive dysphasia. His admission MRI brain showed an acute cortical infarct with vasogenic and cytotoxic edema affecting the precentral gyrus of the left frontal lobe, on Fluid Attenuation Inversion Recovery T2-weighted imaging (Figure 1, black arrows) and diffusion-weighted imaging (Figure 2A and 2B, black arrows). There was no intracranial haemorrhage and this infarct was deemed embolic in origin.

**Figure 1:**
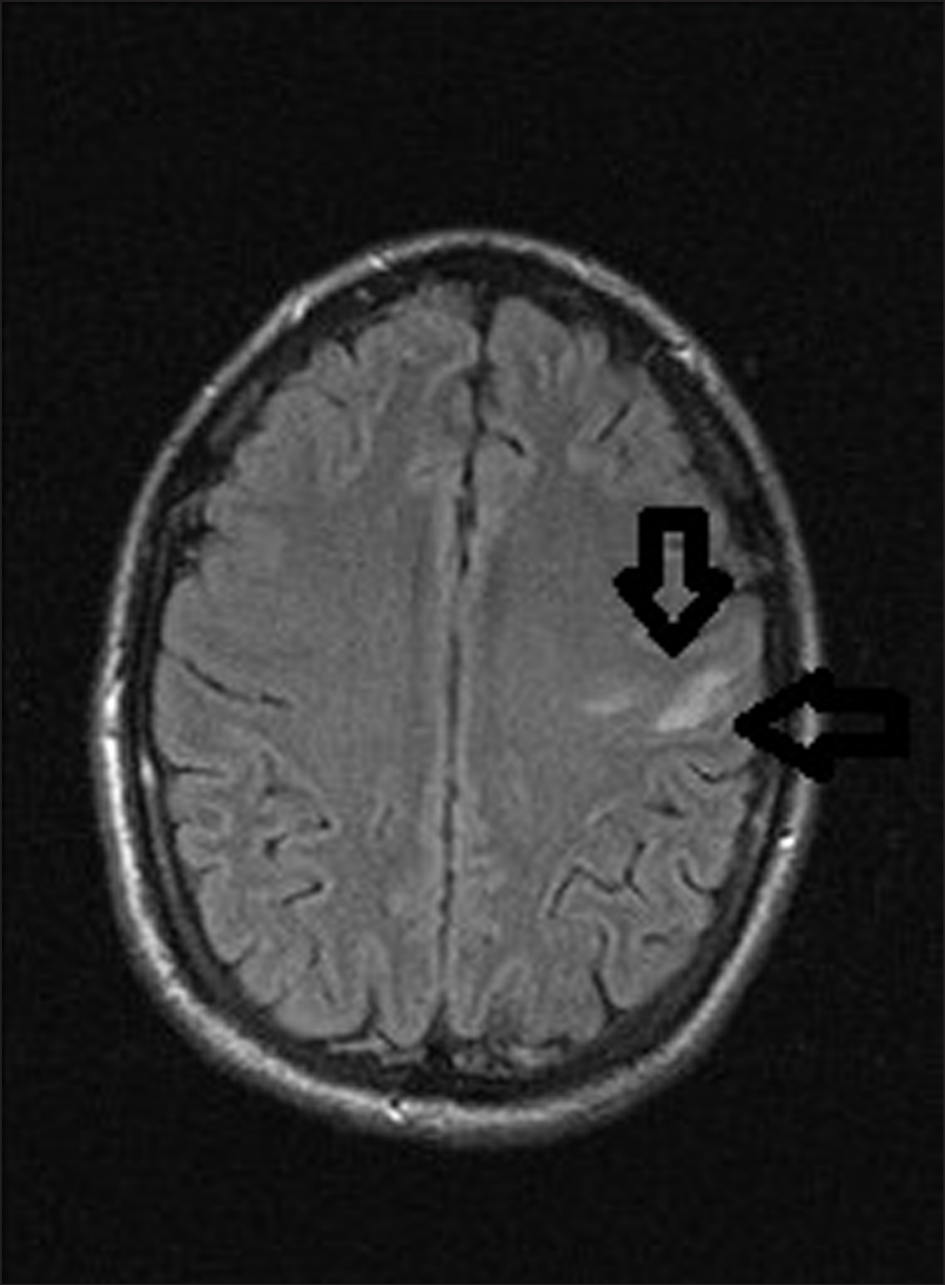


**Figure 2:**
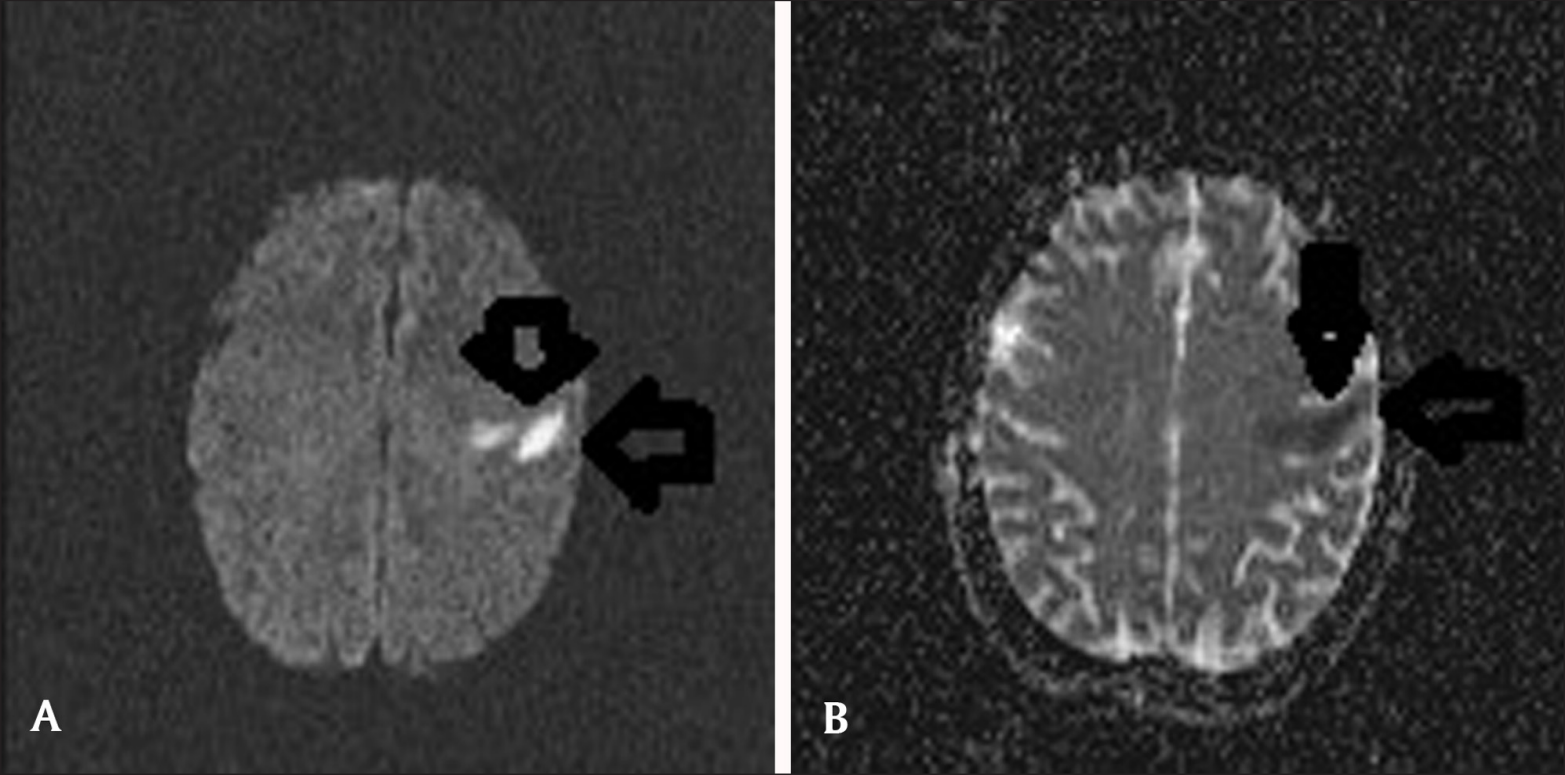


Admission bloods showed an elevated ESR and anaemia, while blood cultures unexpectedly grew Granulicatella adiacens. A transoesophageal echocardiogram showed vegetations involving the mitral and aortic valves. Both valves were repaired and postoperatively he complained of dyspnoea. A CT pulmonary angiogram was negative for pulmonary emboli but revealed multiple large splenic artery aneurysms, a clinically unexpected but significant complication of infective endocarditis (Figure 3A and 3B, black arrows). These were treated with an emergency splenectomy. Based on these imaging finding this was a case on infective endocarditis with extra cardiac complications causing both an embolic stroke and mycotic splenic artery aneurysms.

**Figure 3:**
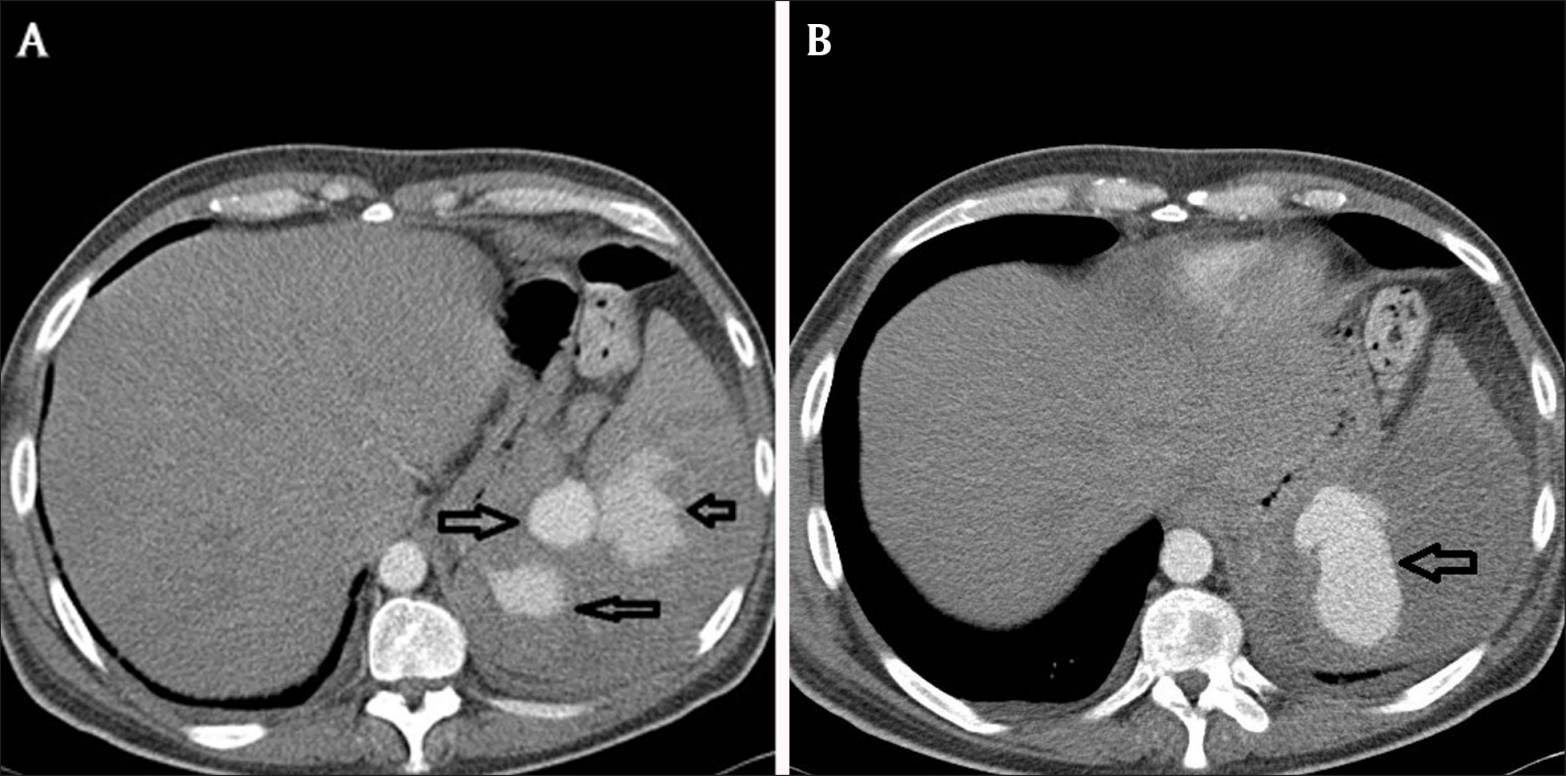


## Comment

Granulicatella adiacens is a nutritionally variant streptococcus. To date 29 cases of infective endocarditis due to Granulicatella species have been reported in the literature. Although this species has frequently been associated with complications of infective endocarditis such as emboli, perivalvular abscess and heart failure, there currently are no reported cases of splenic artery mycotic aneurysms due to Granulicatella. The true incidence of splenic artery aneurysms is not known and quoted range is anything from up to 10.4%. It is the third most common intraabdominal aneurysm after abdominal aorta and iliac arteries, and the commonest visceral aneurysm. The exact aetiology is unknown but unlike aortic aneurysms is not thought to be due to atherosclerosis. Splenic artery aneurysms are associated with conditions affecting the liver or portal venous system including portal hypertension, cirrhosis, liver transplantation, conditions affecting the vessel wall such as arteritis, collagen vascular disease, arterial fibroplasia and inflammatory or infectious disorders.

Most cases like this one are clinically asymptomatic and discovered incidentally on imaging performed for another purpose. Typically when symptoms are present it is due to rupture and presents with abdominal pain and haemodynamic instability. Although an infrequent complication of infective endocarditis if a splenic artery aneurysm ruptured it is a serious and potentially fatal complication. Splenic artery aneurysms are four times more common in females, half of those that rupture are in pregnant females and have a mortality of up to 90%. Treatment is indicated for aneurysms that cause symptoms, more than 30 mm and in pregnant or young females [1].

## Competing Interests

The author declares that they have no competing interests.
